# Multiple COVID-19 Clusters on a University Campus — North Carolina, August 2020

**DOI:** 10.15585/mmwr.mm6939e3

**Published:** 2020-10-02

**Authors:** Erica Wilson, Catherine V. Donovan, Margaret Campbell, Thevy Chai, Kenneth Pittman, Arlene C. Seña, Audrey Pettifor, David J. Weber, Aditi Mallick, Anna Cope, Deborah S. Porterfield, Erica Pettigrew, Zack Moore

**Affiliations:** ^1^North Carolina Division of Public Health; ^2^Epidemic Intelligence Service, CDC; ^3^Orange County Health Department, Hillsborough, North Carolina; ^4^Campus Health, University of North Carolina at Chapel Hill; ^5^Gillings School of Public Health, University of North Carolina at Chapel Hill; ^6^North Carolina Department of Health and Human Services; ^7^CDC COVID-19 Response Team; ^8^University of North Carolina School of Medicine, Chapel Hill.

*On September 29, 2020, this report was posted online as an *MMWR* Early Release.*

Preventing transmission of SARS-CoV-2, the virus that causes coronavirus disease 2019 (COVID-19), in institutes of higher education presents a unique set of challenges because of the presence of congregate living settings and difficulty limiting socialization and group gatherings. Before August 2020, minimal data were available regarding COVID-19 outbreaks in these settings. On August 3, 2020, university A in North Carolina broadly opened campus for the first time since transitioning to primarily remote learning in March. Consistent with CDC guidance at that time (*1*,*2*), steps were taken to prevent the spread of SARS-CoV-2 on campus. During August 3–25, 670 laboratory-confirmed cases of COVID-19 were identified; 96% were among patients aged <22 years. Eighteen clusters of five or more epidemiologically linked cases within 14 days of one another were reported; 30% of cases were linked to a cluster. Student gatherings and congregate living settings, both on and off campus, likely contributed to the rapid spread of COVID-19 within the university community. On August 19, all university A classes transitioned to online, and additional mitigation efforts were implemented. At this point, 334 university A–associated COVID-19 cases had been reported to the local health department. The rapid increase in cases within 2 weeks of opening campus suggests that robust measures are needed to reduce transmission at institutes of higher education, including efforts to increase consistent use of masks, reduce the density of on-campus housing, increase testing for SARS-CoV-2, and discourage student gatherings.

University A students returned to residence halls during August 3–9, 2020, and in-person classes began on August 10. Mitigation steps taken to prevent the spread of SARS-CoV-2 on campus included scheduling move-in appointments across a 1-week period, decreasing classroom density to facilitate physical distancing, and reducing maximum dining hall capacity and increasing takeout options. Students were required to sign an acknowledgment of community standards and university guidelines recommending daily symptom checks, use of masks in all indoor common spaces and classrooms, physical distancing of ≥6 feet in indoor and outdoor settings, and limitations on group gatherings consistent with local guidelines (groups of no more than 10 persons indoors and 25 outdoors). Approximately 95% of students signed the acknowledgment; however, data on adherence to these important mitigation strategies were not available. Reentry testing for COVID-19 and quarantine before or after arrival on campus were not used (*1*). Except for two dormitories reserved for isolation and quarantine, residence halls opened at 60%–85% capacity, with most students in double rooms. Those at increased risk for severe illness from COVID-19, according to CDC guidance (*3*), had the option to request a single room. Undergraduate enrollment in university A for the fall semester was 19,690 students. Approximately 5,800 (29%) of these undergraduate students resided on campus as of August 10. In 2019, 83% of undergraduate students were North Carolina residents.

By August 25, 670 laboratory-confirmed cases of COVID-19 with a specimen collection date for SARS-CoV-2 testing of August 3 or later had been identified among students, faculty, and staff members at university A (Figure). Cases were identified by the student health clinic (by self-report or through testing at the student health clinic or the university hospital testing center) or linked to a university cluster by the local health department. Initial information was collected by the university at the time of testing; the university also implemented contact tracing, isolation, and quarantine. Additional investigation of cases was conducted by the local health department for students who were tested off campus. Cases were classified according to the Council of State and Territorial Epidemiologists COVID-19 2020 Interim Case Definition (*4*). An additional 120 potential cases identified by the student health clinic had insufficient information to meet criteria for confirmed or probable COVID-19 and were not included in the analysis. Information on cases reported only to the university employee occupational health clinic, which is separate from the student health clinic, was not available for review at the time of analysis.

**FIGURE:**
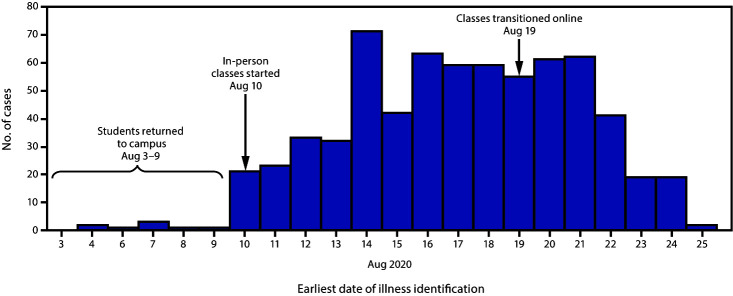
Confirmed COVID-19 cases among university A students, faculty, and staff members (N = 670), by earliest illness identification date — North Carolina, August 2020 **Abbreviation:** COVID-19 = coronavirus disease 2019.

Among 670 confirmed cases with specimen collection dates during August 3–25 for SARS-CoV-2 testing, median patient age was 19 years (range = 17–50 years), and 293 (47%) cases occurred in males (information on gender was missing for 47 [7%] patients). Information on school affiliation (e.g., undergraduate versus graduate/professional student, faculty, or staff member) was not consistently recorded; however, considering patient age <22 years as an indicator of undergraduate status, 643 (96%) cases were estimated to have occurred in undergraduate students; among these students, 230 (36%) resided on campus, and at least 51 (8%) were members of a fraternity or sorority and 51 (8%) were student athletes. For the remainder, place of residence, including if living at home or in shared apartments, was not readily available. As of August 25, no COVID-19 patients were hospitalized or had died, and no cases of multisystem inflammatory syndrome in children or adults were reported. One student was kept for extended observation in a hospital emergency department. Information on other clinical manifestations, such as myocarditis, was not available.

Clusters were defined as the occurrence of five or more epidemiologically linked cases (e.g., common residence, sports team, or fraternal organization membership) within 14 days of one another (by earliest date of illness identification). During August 3–25, 18 clusters at university A were identified, eight in residence halls, five among students with membership in a fraternity or sorority, one in off-campus apartments, and four among athletic teams. Overall, 201 (30%) cases were linked to a cluster. Clusters ranged in size from five to 106 patients (median = five), with the largest cluster associated with a university-affiliated apartment complex.

On August 19, when 334 (50%) university A–associated cases had been reported to the local health department, all university A classes transitioned to online, and efforts to reduce the density of on-campus housing commenced. Testing for SARS-CoV-2 was recommended for all persons living in residence halls with case clusters and was offered to all students at the student health clinic and the university hospital testing center. Students living in on-campus residence halls were required to return home unless they applied for and received a hardship waiver indicating they could remain on campus. All students returning home were instructed to self-quarantine for 14 days following departure from campus. Off-campus testing sites were set up both to meet community needs and target off-campus student housing complexes with multiple cases.

## Discussion

Rapid increases in COVID-19 cases occurred within 2 weeks of opening university A to students. Based on preliminary case investigations, student gatherings and congregate living settings, both on and off campus, likely contributed to the rapid spread of COVID-19 on campus. This suggests the need for robust and enhanced implementation of mitigation efforts and the need for additional mitigation measures specific to this setting.

The findings in this report are subject at least five limitations. First, the number of reported cases at university A is likely an underestimate. For example, some cases were reported to students’ home jurisdictions, some students did not identify themselves as students to the county health department, some students did not report to the student health clinic, and not all students were tested. Second, the number of students possibly infected through affiliation with a fraternity or sorority is likely underestimated. Some students might not have disclosed their fraternity or sorority membership, and other students (who were not members of fraternities or sororities) might have participated in unofficial rush events and parties. Third, limited information was available on housing arrangements for students not identified to live on campus, as well as information about the extent of social gatherings and adherence to masking and other important mitigation efforts. Fourth, cases had limited clinical follow-up; thus, the extent of longer-term clinical complications is not known. Finally, because information available on cases in faculty and staff members was limited, the contribution of faculty or staff members to COVID-19 spread on campus cannot be estimated.

The rapid increase in COVID-19 cases among college-aged persons at university A underscores the urgent need to implement comprehensive mitigation strategies (*5*,*6*). In addition to enforcement of mask requirements, measures needed to reduce transmission in college and university settings might include efforts to reduce the density of on-campus housing, increase testing for SARS-CoV-2, and discourage student gatherings. Emerging findings from ongoing monitoring and evaluation efforts at universities and colleges in North Carolina and nationwide are helping to update best practices, including optimal testing strategies, for preventing SARS-CoV-2 transmission on campus and in the adjacent communities.

**Summary**

**What is already known about this topic?**

Before August 2020, minimal data were available about outbreaks and disease transmission in institutes of higher education within the United States.

**What is added by this report?**

A North Carolina university experienced a rapid increase in COVID-19 cases and clusters within 2 weeks of opening the campus to students. Student gatherings and congregate living settings, both on and off campus, likely contributed to the rapid spread of COVID-19 in this setting.

**What are the implications for public health practice?**

Enhanced measures are needed to reduce transmission at institutes of higher education and could include reducing on-campus housing density, ensuring adherence to masking and other mitigation strategies, increasing testing for SARS-CoV-2, and discouraging student gatherings.
